# Impact of passing mesenchymal stem cells through smaller bore size needles for subsequent use in patients for clinical or cosmetic indications

**DOI:** 10.1186/1479-5876-10-229

**Published:** 2012-11-21

**Authors:** Murali Krishna Mamidi, Gurbind Singh, Juani Mazmin Husin, Kavitha Ganesan Nathan, Gopinath Sasidharan, Zubaidah Zakaria, Ramesh Bhonde, Anish Sen Majumdar, Anjan Kumar Das

**Affiliations:** 1 Stempeutics Research Malaysia Sdn. Bhd, Technology Park Malaysia, 57000, Kuala Lumpur, Malaysia; 2 Manipal Institute of Regenerative Medicine, Manipal University Branch Campus, # 10 Service Road, Domlur Layout, Bangalore, 560071, India; 3 Hematology Unit, Cancer Research Centre, Institute for Medical Research, Jalan Pahang, 50588, Kuala Lumpur, Malaysia; 4 Stempeutics Research Pvt. Ltd, Akshay Tech Park, Whitefield, Bangalore, 560066, India

**Keywords:** Bone Marrow Mesenchymal stem cells (BM-MSC), Differentiation, Cell migration needle bore size, MSC transplantation/infusion

## Abstract

**Background:**

Numerous preclinical and clinical studies have investigated the regenerative potential and the trophic support of mesenchymal stem cells (MSCs) following their injection into a target organ. Clinicians favor the use of smallest bore needles possible for delivering MSCs into vascular organs like heart, liver and spleen. There has been a concern that small needle bore sizes may be detrimental to the health of these cells and reduce the survival and plasticity of MSCs.

**Methods:**

In this report, we aimed to investigate the smallest possible bore size needle which would support the safe delivery of MSCs into various tissues for different clinical or cosmetic applications. To accomplish this we injected cells via needle sizes 24, 25 and 26 G attached to 1 ml syringe in the laboratory and collected the cells aseptically. Control cells were ejected via 1 ml syringe without any needle. Thereafter, the needle ejected cells were cultured and characterized for their morphology, attachment, viability, phenotypic expression, differentiation potential, cryopreservation and *in vivo* migration abilities. In the second phase of the study, cells were injected via 26 G needle attached to 1 ml syringe for 10 times.

**Results:**

Similar phenotypic and functional characteristics were observed between ejected and control group of cells. MSCs maintained their cellular and functional properties after single and multiple injections.

**Conclusions:**

This study proves that 26 G bore size needles can be safely used to inject MSCs for clinical/therapeutics purposes.

## Background

Regenerative medicine is a multidisciplinary, young and emerging field in biotechnology and medicine, which is expected to change patient treatment profoundly, generating and regenerating tissues and organs instead of merely ameliorating symptoms. Stem cells is a branch of regenerative medicine treating damaged tissues by introducing progenitor cells into a tissue or organ and is believed to offer treatment for many degenerative diseases such as Alzheimer’s, Parkinson’s, heart disease, rheumatoid arthritis, osteoarthritis and many others [1]. Mesenchymal stem cells (MSCs) have become a popular source for cell therapy research because they are multipotent with the capability to differentiate into a variety of cell types including osteocytes, chondrocytes, adipocytes and myocytes under specific culture conditions [2]. They were initially characterized by Friedenstein and co-workers more than 40 years ago, and were described as fibroblast-like cells with the property of adhering to plastic in culture [3]. Bone marrow is the conventional source of MSCs; later these cells have been isolated from variety of tissues from head to toe [4-6]. The Mesenchymal and Tissue Stem Cell Committee of the International Society for Cellular Therapy proposed minimal criteria to define human MSCs. As per these criteria MSCs must be plastic-adherent, they must express CD105, CD73 and CD90; lack expression of CD45, CD34, CD14 or CD11b, CD79alpha or CD19 and HLA-DR surface molecules and they must differentiate to osteoblasts, adipocytes and chondroblasts *in-vitro* in response to specific stimuli [7]. MSCs also express wide variety of cell surface and adhesion molecules such as STRO-1, ICAM-1/2, ALCAM-1, L- selectin [8]. MSCs repair the damaged tissues by secreting trophic factors such as chemokines, cytokines, and extracellular matrix proteins [9] apart from their regeneration ability.

The role of stem cells in the clinical field has gathered tremendous momentum over the last decade and MSCs become a focus of interest for use in clinical therapies for various diseases and injuries. Although adult stem cells have been described from a wide range of adult tissues, the well characterized source for adult stem cells is still bone marrow. BM-MSCs are an excellent candidate for cell therapy because (a) they can be easily isolated and expanded to clinical scale in a very short period of time; (b) ease of accessibility; (c) can be biopreserved with minimal loss of stem cell characteristics; (d) immunosuppressive nature and, (e) most importantly, so far human clinical trials of MSCs have shown no adverse reactions in either allogeneic or autologous transplantation scenario [2]. In fact, clinical trials have revealed the feasibility and safety of the clinical use of MSCs and have provided some evidence of efficacy in various medical conditions [10]. Immunomodulatory functions of MSCs make them as an important candidate for the treatment of autoimmune diseases [11] such as rheumatoid arthritis [12], Type 1 diabetes [13] and multiple sclerosis [14,15]. Furthermore, adult stem cells have helped to prevent corneal degeneration and to restore vision in cases of blindness [16]. They have also restored proper cardiac function to heart attack sufferers [17] and improved movement in spinal cord injury patients [18].

Recent guidelines issued by the regulatory body CBER (Center for Biologics Evaluation & Research) suggests that cell therapy products should have 80% viability or more and show a repeatedly high level of potency [19]. Numerous studies have analyzed various factors which could affect the cell viability and other parameters during and after cell delivery [1,20]. Many of the methods of cell delivery require the use of syringes to deliver the cells to the appropriate site, for instance, multiple injections are made to the left ventricular myocardium when treating heart failure due to ischemic disease. While physicians always prefer to use narrow bore needles for the comfort of the patient or to prevent unnecessary bleeding; the narrowed bores may cause damage to the cells during the passage through the needle. The size of the needle could have an effect on cell viability and functional changes could be induced by the stress of expulsion of the suspension from a narrow bored-sized needle. Thus, we designed this study to determine the impact on BM-MSCs while injecting them via different bore-size needles. Further, we also evaluated the effect of repeated injections on BM-MSCs via 26 G bore size needle.

## Material and methods

### MSC isolation and culture

MSCs were obtained from bone marrow samples of healthy donors aged between 20–35 years after obtaining informed consent and the protocol was approved by the institutional ethics committee (Manipal Hospital, Bangalore). MSCs were isolated as reported by us earlier [21]. Briefly, bone marrow mononuclear cells (MNCs) were separated by the Ficoll density gradient method (1.077 g/ml density) in 50 ml centrifuge tubes (Falcon, Becton-Dickinson). Bone marrow MNCs accumulated on the Ficoll–plasma interface were isolated and washed again with KO-DMEM. Isolated cells were plated into T-75 cm^2^ culture flasks (Falcon, Becton-Dickinson) and cultured in KO-DMEM supplemented with 10% fetal bovine serum (FBS; HyClone), 2 mM glutamax and Pen-Strep (Gibco–Invitrogen) and incubated at 37°C and 5% humidified CO_2_. Cells were supplemented with fresh media every 48 h and upon confluency, the cells were harvested with 0.25% trypsin–EDTA (Gibco–Invitrogen) and re-plated in suitable tissue culture dishes for expansion.

### Injection of MSCs via different bore size needles

MSCs were injected via different bore size needles such as 24, 25 and 26 G (all needles from Becton & Dickinson) attached to 1 ml syringe with the flow rate of 2000 microl/min. Control group of cells were passed through the 1 ml syringe without needle and cultured at 37°C and 5% Co_2_ incubator (Binder). During the second phase of the study, cells were continuously injected for 10 repeats (multiple injections) via 26 G needle. The MSCs ejected for 2^nd^, 4^th^, 6^th^, 8^th^ & 10^th^ time were characterized for morphology, viability, phenotypic expression and differentiation potential to study the effect of multiple injections on MSCs.

### Cryopreservation and resuscitation

MSCs were re-suspended in freezing solution containing 90% (v/v) sterile FBS and 10% (v/v) dimethylsulfoxide (DMSO; Sigma). Cells were loaded in 2 ml cryovials (Nunc) at a concentration of 3 × 10^6^ cells/vial and frozen using a programmable slow freezing unit (Planar Kryo 560-16). After freezing, the cryovials were transferred in a liquid nitrogen vapour-phased cryo-container (Statebourne Cryogenics; BioSystem 36) for long term storage. The frozen stocks were thawed in a constant-temperature water bath at 37°C by shaking lightly. After 1 or 2 min, cells were re-suspended in complete medium and centrifuged at 1800 r.p.m. for 10 min. MSCs were thawed and injected through 26 G needle and analyzed for percentage of cell viability, stromal marker expression and remaining cells were cultured to study their differentiation potential.

### CM-DiI labeling of MSCs

For detection of MSCs *in-vivo*, we have labeled the cells using CM-DiI fluorescent dye (invitrogen). Cells were removed and centrifuged at 1200 rpm for 10 min and the supernatant was discarded. 25 million cells were re-suspended in KO-DMEM (Gibco) along with 4 μM of CM-DiI dye and incubated for 30 min at 37°C. Cells were washed twice with KO-DMEM to remove the unbound dye. The cell pellet was re-suspended in 500 μL of DPBS (invetrogen) for subsequent *in-vivo* transplantation and tracking studies.

### *In-vivo* injection and tracking of DiI labeled MSCs

Eight-ten weeks old Nude rats were used in our studies with prior ethical approvals and all animal procedures were performed in accordance with our institutional guidelines. Animals were anesthetized by using isoflurane in the induction chamber of *In Vivo* Imaging System (IVIS; Caliper Life Sciences) and the cells were delivered intravenously (i.v) by tail vein injection. Then the animals were placed in the IVIS imaging chamber which consists of a supersensitive cooled charge-coupled device (CCD) camera mounted inside a light-tight imaging chamber. The gray scale photographic and fluorescent images were superimposed using the Living Image V 4.2 software overlay (Caliper Life Sciences).

### Determination of viability

Cell viability was assessed by 7-amino actinomycin D (7-AAD) staining using flow cytometry as described earlier [5].

### Immunophenotyping by flow cytometry analysis

MSCs were harvested and resuspended in PBS at a cell density of 1.0 × 10^6^ cells/ml. Two hundred microliters of the cell suspension (approximately 1 × 10^5^ cells) was incubated with the labeled antibodies in dark for 30 min at room temperature (RT). The following antibodies were used to mark the cell surface epitopes-CD90-phycoerythrin (PE), CD44-PE, CD73-PE, CD166-PE and CD34-PE, CD45-fluoroisothyocyanate (FITC), and HLA-DR-FITC (all from BD Pharmingen, San Diego, CA). At least 10,000 events were acquired on Guava Technologies flow cytometer, and the results were analyzed using Cytosoft, Version 5.2, Guava Technologies, Hayward, CA.

### Determination of cell senescence by β-galactosidase assay

Senescence assay was performed with the MSCs cultured after injection via different bore size needles using Senescence β-Galactosidase Staining kit (Cell Signaling Technologies, Danvers, MA, USA,) according to the manufacturer’s protocol. Senescent cells were identified as blue-stained cells by standard light microscopy, and a minimum of 100 cells were counted in 10 random fields to determine the percentage of SA-ß-galactosidase-positive cells [22].

### Differentiation potential of MSC

To assess the mesodermal differentiation potential, MSCs were cultured at a density of 1000 cells/cm^2^ in six well plates (Nunc) and were allowed to reach confluence. Differentiation potential of MSCs towards osteogenic, chondrogenic and adipogenic potential was assessed using published protocols [5].

### Statistical analysis

All the experiments were replicated three times (*n* = 3). Data was presented as mean ± SEM, and results were analyzed by student *t*-test. Differences were considered statistically significant when *P* <0.05.

## Results

### Effect of different bore size needles on MSC characteristics

**Figure 1:**
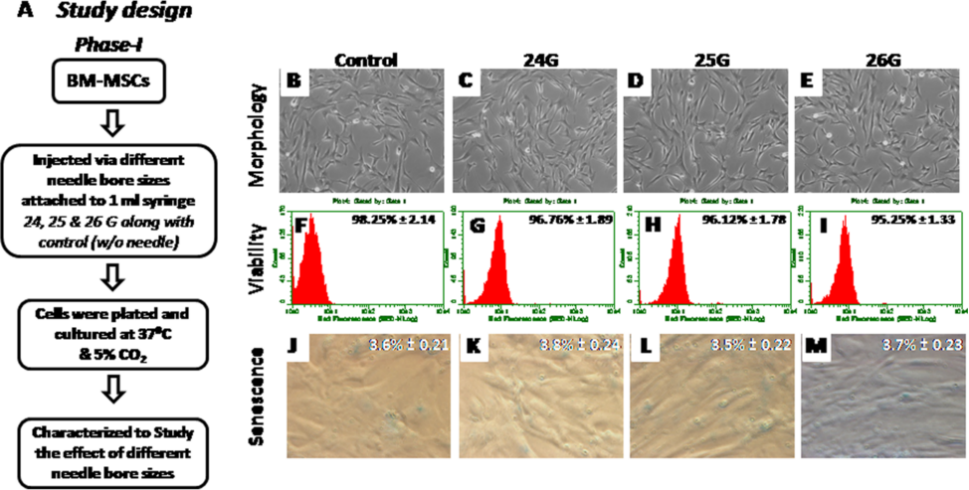
**Characterization of BM-MSCs injected via different bore size needles: (A) Describes phase I study design flow chart.** (**B** - **E**) Represent the morphology; (**F** - **I**) viability; and (**J** - **M**) senescence studies of BM-MSCs injected via different bore size needles ranging from 24 G to 26 G respectively along with the control cells injected via syringe with our any needle.

We conducted the entire study in two phases to identify the smallest bore size needle for the safe delivery of MSCs. During the first phase of the study, cells were injected through different bore size needles (24 G, 25 G and 26 G) and characterized them in comparison with control cells (Figure 1 A). We showed that single injection of cells via 26 G was safe and non detrimental to the biology of the cell. In the next phase, we checked the effect of multiple injections of MSCs via 26 G bore size needle.

### Effect of needle bore sizes on cell attachment, morphology and viability of MSCs after injection

Cells were plated in 6 well tissue culture dishes after injected through variety of bore size needles such as 24 G, 25 G and 26 G to examine their attachment pattern and cell morphology. All the injected group of cells attached within 24 hours of plating and showed normal spindle shape MSCs morphology similar to that of control cells (Figure 1B-E). High percentage of viability was retained for the ejected group of cells through different needle gauges. Percentage of cell viability of injected cells via different bore sizes such as 24 G, 25 G and 26 G was compared with the viability of control cells which were injected via syringe without attached to any bore size needles (Figure 1F-I). No significant differences were observed between the injected groups when compared with control group of cells. During the second phase of the study, percentage of cell viability remains virtually same for all multiple injections (Table 1).

**Table 1:** Viability and phenotype of BM-MSCs ejected via 26 G bore size needle for multiple times

	**Control**	**2**^**nd**^**Jab**	**4**^**th**^**Jab**	**6**^**th**^**Jab**	**8**^**th**^**Jab**	**10**^**th**^**Jab**
**Viability**	98.25% ± 1.45	96.98% ± 2.11	97.38% ± 2.23	95.89% ± 1.89	96.18% ± 2.19	97.35% ± 1.95
**CD44**	98.68% ± 1.42	98.89% ± 1.18	98.13% ± 1.56	98.54% ± 1.39	98.72% ± 1.28	98.31% ± 1.49
**CD73**	98.97% ± 1.16	98.63% ± 1.36	98.48% ± 1.41	98.77% ± 1.29	98.22% ± 1.67	98.41% ± 1.37
**CD166**	96.48% ± 2.24	97.12% ± 2.16	96.29% ± 2.31	97.71% ± 2.24	97.11% ± 2.18	96.89% ± 2.29
**CD34**	0.02%	0.23%	0.76%	0.94%	0.68%	0.85%
**CD45**	0.06%	0.66%	0.47%	0.57%	0.81%	0.90%
**HLA-DR**	0.05%	0.11%	0.76%	0.84%	0.93%	0.18%

### MSC senescence after injecting through different bore size needles

The enzyme lysosomal pH6 β-galactosidase (SA-β-gal) was employed as a senescence marker to look at the percentage of cells undergoing senescence after injection. Very few cells were found to express SA- β-gal stain, suggesting that these cells were not damaged during the injection process. Moreover, we observed a very small percentage of SA-β-gal positivity for all injected group of cells when compared with controls (Figure 1J-M). These results indicate that there were no significant differences observed for both manipulated and control group of cultures.

### Surface phenotype characterization

**Figure 2:**
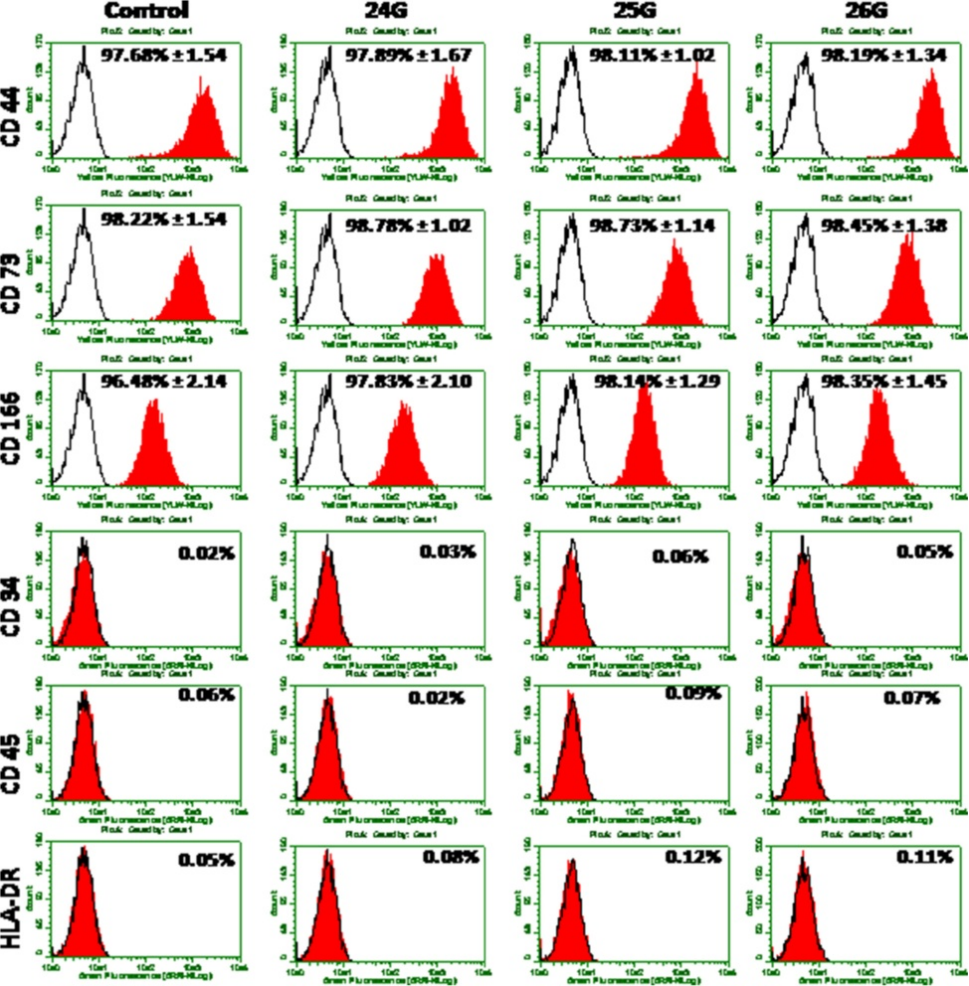
**Detection of surface marker expression of BM-MSCs: Phenotypic expression of BM-MSCs after ejected via different bore size needles along with control cells were analyzed by flow cytometry.** An open area represents an antibody isotype control for background fluorescence and a shaded area shows signal from MSC surface marker antibodies. Representative histograms are depicted.

Flow cytometry analysis revealed stromal marker expression of injected and control group of cells. Cells injected via different bore size needles showed the positive expression of the MSC markers CD44, CD73 and CD166 and negative for CD34, CD45 and HLA-DR similar to that of the un-manipulated cells (Figure 2). Phase two study disclosed insignificant differences among the stromal marker expression for multiple injections. Cells ejected for multiple times retained their high percentage of stromal marker expression (Table 1). As expected, the expression of CD34, CD45 and HLA-DR was found to be negative in all cell groups confirmed their mesenchymal nature.

### Differentiation potential of MSCs after injection

**Figure 3:**
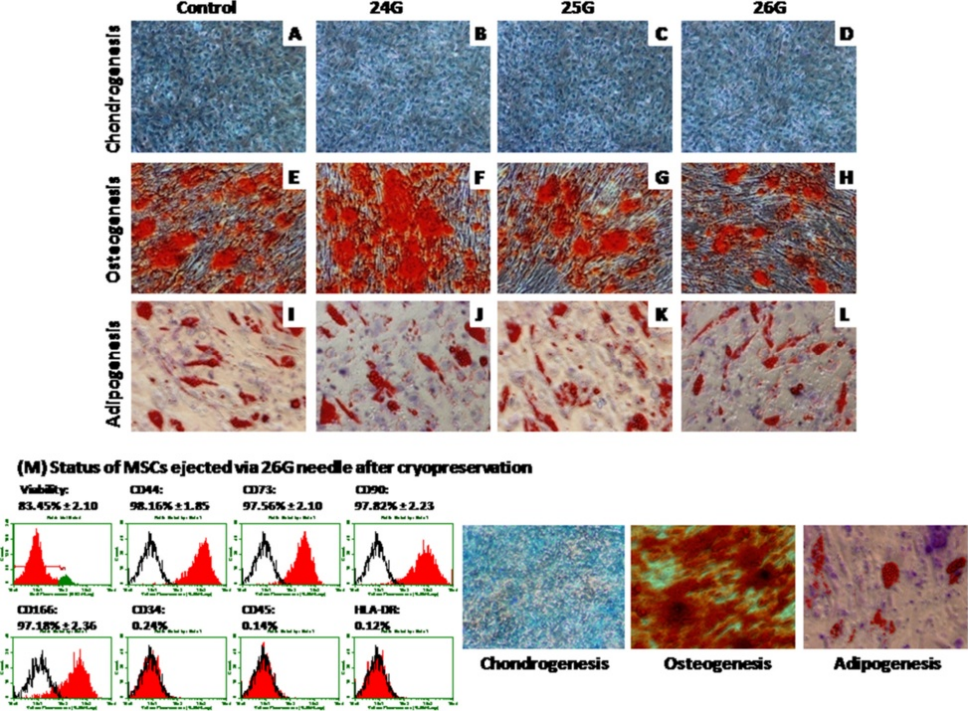
**Differentiation studies: Multilineage differentiation potential of cultured BM-MSCs injected via different bore size needles along with controls.** (**A** - **D**) Chondrogenic differentiation potential was demonstrated by Alcian blue staining. (**E** - **H**) Formation of mineralized matrix was detected by alizarin Red staining confirms the osteogenic differentiation. (**I** - **L**) Adipogenesis was confirmed by neutral oil droplet formation stained with Oil Red O. (M) Represents viability, stromal marker expression and mesodermal tri-lineage differentiation potential of MSCs ejected through 26 G needle after cryopreservation.

**Figure 4:**
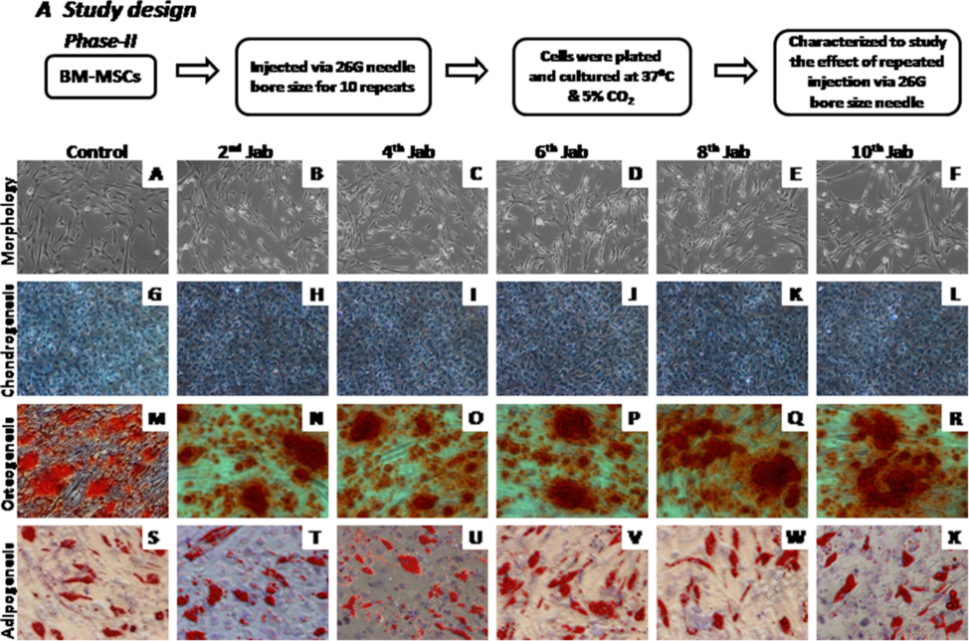
**Morphology and differentiation potential of BM-MSCs injected for multiple times via 26 G: (A) Represents phase II flow chart study design**. (**B** - **F**) Indicates the morphology of BM-MSCs after multiple injections. (**G** - **L**) Represents chondrogenesis; (**M** - **R**) osteogenesis; (**S** - **X**) Adipogenesis of BM-MSCs injected for multiple times via 26 G bore size needle.

Multi-lineage differentiation into chondro-, adipo- and osteogenic potential was analyzed for cells ejected for single injection via 24 G, 25 G and 26 G bore size needles and multiple injections via 26 G bore size needle. Alcian blue staining was used to assess the formation of proteoglycans, which confirmed chondrogenic differentiation ability for single and multiple injections (Figures 3A-D, 4G-L). Cytoplasmic inclusions of neutral lipid vacuoles after adipogenic differentiation were stained with Oil Red O for both single and multiple injected BM-MSCs (Figures 3I-L, 4S-X). Similarly, calcium deposits characteristic for osteogenic differentiation in cultures were visualized by Alizarin red staining for both groups (Figure 3E-H, 4M-R). Hence, we conclude that even after single or multiple injections, BM-MSCs retained their potential to differentiate *in vitro* towards osteo-, chondro-and adipogenic lineages.

### MSCs ejected through 26 G needle after cryopreservation and resuscitation retains their phenotypic expression and differentiation potential

MSCs after thawing injected via 26 G needle and were analyzed for their viability, stromal marker expression and their tri-lineage mesodermal differentiation potential. Although there was slight reduction in cell viability, the expression of cell surface markers and differentiation potential into chondrogenesis, osteogenesis and adipogenesis of these cryopreserved MSCs (Figure 3M; 4) were comparable with that of the control MSCs. These results clearly favor the applicability of this technology for cryopreserved MSCs and their subsequent clinical/therapeutic use.

### *In-vivo* migration of CM-DiI labeled MSCs

**Figure 5:**
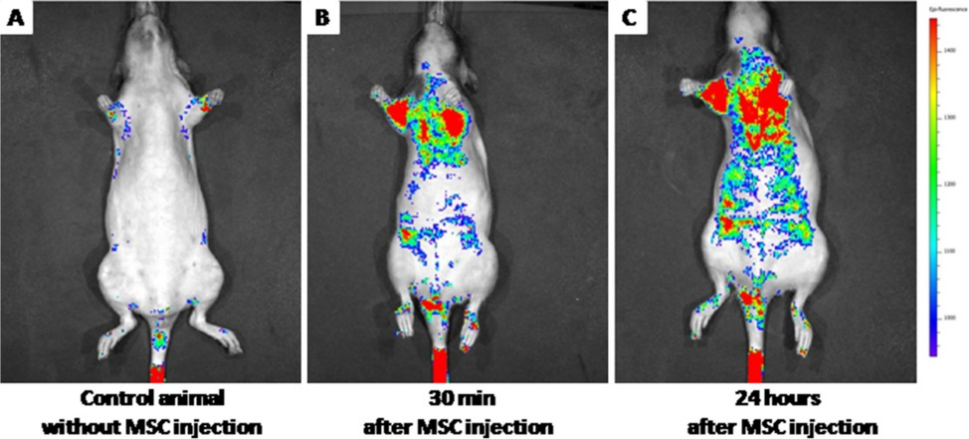
In-vivo migration of BM-MSCs injected into the tail vein using 26 G needle: (A) Represents control animal without cell injection; (B) Indicates migration pattern of BM-MSCs after 30 minutes; (C) Represents grater migration ability of BM-MSCs after 24 hours of implantation.

The CM-DiI labeled MSCs were injected into the tail vein of the Nude rats using 26 G needle. In the control animal without any cell injection, we did not observe any signal except at the tail region (Figure 5A). This auto fluorescence is because of the scales surrounding the tail region of the Nude rats and this signal was persistent throughout the study. However, 30 minutes after injecting 25 million labeled cells into the tail vein of the Nude rate, we saw a distinguishable signal at the upper thoracic region over and above the control animal (Figure 5B). Subsequently after 24 hours we observed detectable greater migration of cells towards the abdomen and to the lower limb regions (Figure 5C). These data sets clearly suggest even in the *in-vivo* condition, MSCs injected through 26 G needle maintain their integrity and were able to migrate to different parts of the healthy animal.

## Discussion

BM-MSCs have generated great excitement in the field of regenerative medicine and are being investigated to treat a wide variety of medical conditions. The route of delivery is of essential importance for the outcome of clinical trials using BM-MSCs. While physicians prefer to use the narrowest bore needles for injection in order to reduce patient discomfort and pain as well as to reduce oozing from the injection site, biologists have concerns about the effect of the injection procedure on the viability and biological activity of the cells. The main concerns of cell delivery through very fine needles is that the cells may not survive the shear force, and the larger is the cell size, the more difficult it is for the cells to survive after ejection from the needle. MSCs on average are usually between 8 – 20 microns in size which is significantly larger compared to hematopoietic stem cells or lymphocytes [23]. This raises the concerns among the clinician community that MSCs could be damaged if they are injected via small guage needles for direct subcutaneous or intramuscular delivery into the skin, muscle tissues and vascular organs like heart, liver and spleen or i.v delivery. Our study clearly demonstrates that MSCs could be successfully delivered into these tissues using small guage needles without hampering the biological activity of these cells. Some conditions, including ischemic heart disease and critical limb ischemia, local injection or even multiple injections is required, therefore, the safety of the cells plus needle as a “combined product” causes much concern among regulators and clinical investigators. In this manuscript, we have reported the viability, senescence, surface marker, tri-lineage *in vitro* differential potential, cryopreservation and *in vivo* tracking of BM-MSCs which were injected through small gauge needles and those undergoing multiple injections, providing evidence that BM-MSCs are robust and remain fit after needle injection.

Although the clinical applications of cell therapy are still in their infancy, there is an urgent need to determine safe delivery systems where these cells retain their viability and biological functionality [24]. There have been a few recent studies investigating the actual effect of cell suspension passing through a needle based delivery device; for example, Kondziolka and coworkers assessed the viability of neuronal cells passed through a 25 -gauge needle and cannula using a simple trypan blue exclusion method [25]. Heng and co-workers previously reported the effects of injection through 26-Gauge Nitinol needle at different flow rate on MSCs [26]. In the current study we have clearly demonstrated that the BM-MSCs retain their viability and biological function after injecting through different bore size needles.

We also examined the status of 26 G needle injected MSCs after cryopreservation. Though there was a slight drop of cell viability, our data clearly demonstrated that the 26 G needle ejected MSCs were able to maintain their stromal phenotypes and differentiation potential after cryopreservation and subsequent thawing (Figure 3M). Our earlier reports and studies by other research groups have showed that there would be 10 - 20% reduction in post thaw of cell viability [27,28]. In consistence with these reports, here we have witnessed similar drop of cell viability after cryopreservation and thawing of 26 G needle ejected MSCs.

In certain indications of cell therapy such as the multiple intramuscular injections for critical limb ischemia, repeated intra-muscular injections are made from the same syringe. This study also attempted to mimic this clinical condition as well. We also aimed to find out the clinically relevant smallest possible bore size needles which can be used for safe delivery of cells either by single or multiple injections. We assessed the viability of hMSCs following their ejection through three different clinically relevant bore size needle gauges; 24, 25 and 26 G. The selection of an appropriate and safe needle gauge used during a cell therapy application very much depends on post delivery cellular and functional characteristics, namely the viable cell density, phenotypic expression of the mesenchymal stromal markers, cell senescence and the functional properties such as differentiation of MSCs into mesoderm lineage. As such, cells injected through various needle gauge sizes ranging from 24 to 26 G, have successfully demonstrated their cellular and functional properties. During the second phase of the study, cells were injected through 26 G bore size needle multiple times to mimic certain clinical usage as described above. Cells injected multiple times via 26 gauge bore size needle have also been shown to retain their cellular and functional characteristics.

Since the clinical success of stem cell therapy, in other words restoration of function, tissue integration and/or cell localization [29] is based upon the post transplantation response, the quality of cells injected are an important determinant of the clinical response. Thus, we were interested to see the *in-vivo* migration of MSCs after injecting trough the smallest bore size needle. Earlier researchers demonstrated *in-vivo* migration ability of MSCs [30,31]; however it was not clear about the needle size used to deliver the cells. Here we have showed that the MSCs injected via 26 G needle were safe and migrated to various organs (Figure 5B-C). It has been shown by other research groups that the MSC will migrate to the thoracic cavity immediately after implantation [32,33]. In consistency with these studies we also witnessed similar migration pattern where the MSCs immediately migrated to the thoracic cavity after 30 minutes of injection (Figure 5B) and subsequently to the abdominal cavity and towards the lower limbs after 24 hours (Figure 5C) of implantation. These results confirm that cells were healthy and able to migrate all over the body in the normal healthy animal safely after injecting through 26 G needle. Earlier most of the researches applied the IVIV system to identify *in-vivo* migratory properties of tumor cells in cancer biology. However, here we have successfully demonstrated the usage of IVIS system for studying *in-vivo* MSC migration pattern.

### Conclusion and future perception

Although still in its infancy, cell therapy holds huge promise for the treatment of many diseases with unmet medical needs. It has already been demonstrated by many researchers and clinical trials that the delivery of cells is possible using conventional delivery systems, which is via direct injection *in situ*. We have clearly demonstrated that there is no significant difference in viability and cellular responses caused by the delivery system after post-ejection via different bore size needles up to a minimum of 26 G. This study also further highlights that multiple injections of cells immediately to the desired site is also safe and non detrimental for the cells. Most clinicians would be comfortable with the use of 26 G needles for most clinical purposes. This makes it possible to use direct needle injection into even vascular organs, thus in future therapies, possible injections to the heart muscle, liver, pancreas or even the spleen would also be safe and non injurious to the cells.

## Abbreviations

BM-MSC: Bone Marrow Mesenchymal stem cells; CBER: Center for Biologics Evaluation & Research.

## Competing interests

The authors declare that they have no competing interests.

## Authors’ contributions

MKM conducted all functional and binding experiments, unless otherwise noted, analyzed the results and manuscript writing. G. Sing, JMH & ZZ co-ordinated and conducted *in-vivo* animal experiments and analyzed the data. KGN conducted cell culture experiments. RB contributed to the interpretation of results and editing of the manuscript. GS conducted the flow cytometry experiments and analysis of these data. ASM coordinated design of the experiments, contributed to the interpretation of structural data and drafting the manuscript. AKD conceived the study, coordinated design of the experiments, and drafted the manuscript with assistance as noted above. All authors read and approved the final manuscript.
